# From Knee Pain Consultation to Pituitary Surgery: The Challenge of Cushing Disease Diagnosis

**DOI:** 10.1210/jcemcr/luae048

**Published:** 2024-04-10

**Authors:** Maria Alejandra Gómez-Gutiérrez, Juan Miguel Huertas-Cañas, Andrés Bedoya-Ossa

**Affiliations:** Faculty of Medicine, Pontificia Universidad Javeriana, Bogotá, 110231, Colombia; Faculty of Medicine, Pontificia Universidad Javeriana, Bogotá, 110231, Colombia; Department of Physiological Sciences, Faculty of Medicine, Pontificia Universidad Javeriana, Bogotá, 110231, Colombia

**Keywords:** case report, pituitary ACTH hypersecretion, Cushing syndrome, obesity

## Abstract

Cushing syndrome (CS) is a rare endocrinological disorder resulting from chronic exposure to excessive cortisol. The term *Cushing disease* is used specifically when this is caused by excessive secretion of adrenocorticotropic hormone (ACTH) by a pituitary tumor, usually an adenoma. This disease is associated with a poor prognosis, and if left untreated, it has an estimated 5-year survival rate of 50%. We present the case of a 66-year-old female patient who received a referral to endocrinology for an evaluation of obesity due to right knee arthropathy. Taking into consideration her age, she was screened for osteoporosis, with results that showed diminished bone density. Considering this, combined with other clinical features of the patient, suspicion turned toward hypercortisolism. Laboratory findings suggested that the CS was ACTH-dependent and originated in the pituitary gland. After a second look at the magnetic resonance imaging results, a 4-mm lesion was identified on the pituitary gland, prompting a transsphenoidal resection of the pituitary adenoma.

## Introduction

Chronic excessive exposure to glucocorticoids leads to the diverse clinical manifestations of Cushing syndrome (CS), which has an annual incidence ranging from 1.8 to 3.2 cases per million individuals [1]. The syndrome's signs and symptoms are not pathognomonic, and some of its primary manifestations, such as obesity, hypertension, and glucose metabolism alterations, are prevalent in the general population [2], making diagnosis challenging. Endogenous CS falls into 2 categories: adrenocorticotropic hormone (ACTH)-dependent (80%-85% of cases), mostly due to a pituitary adenoma, or ACTH-independent (15%-20% of cases), typically caused by adrenal adenomas or hyperplasia [3]. Cushing disease (CD) represents a specific form of CS, characterized by the presence of an ACTH-secreting pituitary tumor [1]. Untreated CD is associated with high morbidity and mortality compared to the general population [1], with a 50% survival rate at 5 years [2]. However, surgical removal of a pituitary adenoma can result in complete remission, with mortality rates similar to those of the general population [2]. This article aims to highlight the challenges of suspecting and diagnosing CD and to discuss the current management options for this rare condition.

## Case Presentation

A 66-year-old woman received a referral to endocrinology for an evaluation of obesity due to right knee arthropathy. During physical examination, she exhibited a body mass index of 34.3 kg/m^2^, blood pressure of 180/100, a history of non-insulin-requiring type 2 diabetes mellitus with glycated hemoglobin (HbA1c) of 6.9% (nondiabetic: < 5.7%; prediabetic: 5.7% to 6.4%; diabetic: ≥ 6.5%) and hypertension. Additionally, the patient complained of proximal weakness in all 4 limbs.

## Diagnostic Assessment

Upon admission, densitometry revealed osteoporosis with T scores of −2.7 in the lumbar spine and −2.8 in the femoral neck. Hypercortisolism was suspected due to concomitant arterial hypertension, central obesity, muscle weakness, and osteoporosis. Physical examination did not reveal characteristic signs of hypercortisolism, such as skin bruises, flushing, or reddish-purple striae. Late-night salivary cortisol (LNSC) screening yielded a value of 8.98 nmol/L (0.3255 mcg/dL) (reference value [RV] 0.8-2.7 nmol/L [0.029-0.101 mcg/dL]) and ACTH of 38.1 pg/mL (8.4 pmol/L) (RV 2-11 pmol/L [9-52 pg/mL]). A low-dose dexamethasone suppression test (LDDST) was performed (cutoff value 1.8 mcg/dL [49 nmol/L]), with cortisol levels of 7.98 mcg/dL (220 nmol/L) at 24 hours and 20.31 mcg/dL (560 nmol/L) at 48 hours. Subsequently, a high-dose dexamethasone suppression test (HDDST) was conducted using a dose of 2 mg every 6 hours for 2 days, for a total dose of 16 mg, revealing cortisol levels of 0.0220 nmol/L (0.08 ng/mL) at 24 hours and 0.0560 nmol/L (0.0203 ng/mL) at 48 hours, alongside 24-hour urine cortisol of 0.8745 nmol/L (0.317 ng/mL) (RV 30-145 nmol/24 hours [approximately 11-53 μg/24 hours]) [4].

These findings indicated the presence of endogenous ACTH-dependent hypercortisolism of pituitary origin. Consequently, magnetic resonance imaging (MRI) was requested, but the results showed no abnormalities. Considering ectopic ACTH production often occurs in the lung, a high-resolution chest computed tomography scan was performed, revealing no lesions.

## Treatment

Upon reassessment, the MRI revealed a 4-mm adenoma, prompting the decision to proceed with transsphenoidal resection of the pituitary adenoma.

## Outcome and Follow-Up

The histological analysis revealed positive staining for CAM5.2, chromogranin, synaptophysin, and ACTH, with Ki67 staining at 1%. At the 1-month follow-up assessment, ACTH levels were 3.8 pmol/L (17.2 pg/mL) and morning cortisol was 115.8621 nmol/L (4.2 mcg/dL) (RV 5-25 mcg/dL or 140-690 nmol/L). Somatomedin C was measured at 85 ng/mL (RV 70-267 ng/mL) and prolactin at 3.5 ng/mL (RV 4-25 ng/mL). At the 1-year follow-up, the patient exhibited a satisfactory postoperative recovery. However, she developed diabetes insipidus and secondary hypothyroidism. Arterial hypertension persisted. Recent laboratory results indicated a glycated hemoglobin (HbA1c) level of 5.4%. Medications at the time of follow-up included prednisolone 5 milligrams a day, desmopressin 60 to 120 micrograms every 12 hours, losartan potassium 50 milligrams every 12 hours, and levothyroxine 88 micrograms a day.

## Discussion

CD is associated with high mortality, primarily attributable to cardiovascular outcomes and comorbidities such as metabolic and skeletal disorders, infections, and psychiatric disorders [1]. The low incidence of CD in the context of the high prevalence of chronic noncommunicable diseases makes early diagnosis a challenge [2]. This case is relevant for reviewing the diagnostic approach process and highlighting the impact of the availability bias, which tends to prioritize more common diagnoses over rare diseases. Despite the absence of typical symptoms, a timely diagnosis was achieved.

Once exogenous CS is ruled out, laboratory testing must focus on detecting endogenous hypercortisolism to prevent misdiagnosis and inappropriate treatment [5]. Screening methods include 24-hour urinary free cortisol (UFC) for total cortisol load, while circadian rhythm and hypothalamic-pituitary-adrenal (HPA) axis function may be evaluated using midnight serum cortisol and LNSC [5]. An early hallmark of endogenous CS is the disruption of physiological circadian cortisol patterns, characterized by a constant cortisol level throughout the day or no significant decrease [2]. Measuring LNSC has proven to be useful in identifying these patients. The LNSC performed on the patient yielded a high result.

To assess HPA axis suppressibility, tests such as the overnight and the standard 2-day LDDST [5] use dexamethasone, a potent synthetic corticosteroid with high glucocorticoid receptor affinity and prolonged action, with minimal interference with cortisol measurement [6]. In a normal HPA axis, cortisol exerts negative feedback, inhibiting the secretion of corticotropin-releasing-hormone (CRH) and ACTH. Exogenous corticosteroids suppress CRH and ACTH secretion, resulting in decreased synthesis and secretion of cortisol. In pathological hypercortisolism, the HPA axis becomes partially or entirely resistant to feedback inhibition by exogenous steroids [5, 6]. The LDDST involves the administration of 0.5 mg of dexamethasone orally every 6 hours for 2 days, with a total dose of 4 mg. A blood sample is drawn 6 hours after the last administered dose [6]. Following the LDDST, the patient did not demonstrate suppression of endogenous corticosteroid production.

After diagnosing CS, the next step in the diagnostic pathway involves categorizing it as ACTH-independent vs ACTH-dependent. ACTH-independent cases exhibit low or undetectable ACTH levels, pointing to adrenal origin. The underlying principle is that excess ACTH production in CD can be partially or completely suppressed by high doses of dexamethasone, a response not observed in ectopic tumors [6]. In this case, the patient presented with an ACTH of 38.1 pg/mL (8.4 pmol/L), indicative of ACTH-dependent CD.

Traditionally, measuring cortisol levels and conducting pituitary imaging are standard practices for diagnosis. Recent advances propose alternative diagnostic methods such as positron emission tomography (PET) scans and corticotropin-releasing factor (CRF) tests [7]. PET scans, utilizing radioactive tracers, offer a view of metabolic activity in the adrenal glands and pituitary region, aiding in the identification of abnormalities associated with CD. Unfortunately, the availability of the aforementioned tests in the country is limited.

Once ACTH-dependent hypercortisolism is confirmed, identifying the source becomes crucial. A HDDST is instrumental in distinguishing between a pituitary and an ectopic source of ACTH overproduction [2, 6]. The HDDST involves administering 8 mg of dexamethasone either overnight or as a 2-day test. In this case, the patient received 2 mg of dexamethasone orally every 6 hours for 2 days, totaling a dose of 16 mg. Simultaneously, a urine sample for UFC is collected during dexamethasone administration. The HDDST suppressed endogenous cortisol production in the patient, suggesting a pituitary origin.

In ACTH-dependent hypercortisolism, CD is the predominant cause, followed by ectopic ACTH syndrome and, less frequently, an ectopic CRH-secreting tumor [3, 5]. With the pretest probability for pituitary origin exceeding 80%, the next diagnostic step is typically an MRI of the pituitary region. However, the visualization of microadenomas on MRI ranges from 50% to 70%, requiring further testing if results are negative or inconclusive [5]. Initial testing of our patient revealed no pituitary lesions. Following a pituitary location, ACTH-secreting tumors may be found in the lungs. Thus, a high-resolution chest computed tomography scan was performed, which yielded negative findings. Healthcare professionals must keep these detection rates in mind. In instances of high clinical suspicion, repeating or reassessing tests and imaging may be warranted [3], as in our case, ultimately leading to the discovery of a 4-mm pituitary adenoma.

It is fundamental to mention that the Endocrine Society Clinical Practice Guideline on Treatment of CS recommends that, when possible, all patients presenting with ACTH-dependent CS and lacking an evident causal neoplasm should be directed to an experienced center capable of conducting inferior petrosal sinus sampling to differentiate between pituitary and nonpituitary or ectopic cause [8]. However, in this instance, such a referral was regrettably hindered by logistical constraints.

Regarding patient outcomes and monitoring in CD, there is no consensus on defining remission criteria following tumor resection. Prolonged hypercortisolism results in suppression of corticotropes, resulting in low levels of ACTH and cortisol after surgical intervention. Typically, remission is identified by morning serum cortisol values below 5 µg/dL (138 nmol/L) or UFC levels between 28 and 56 nmol/d (10-20 µg/d) within 7 days after surgical intervention. In our case, the patient's morning serum cortisol was 115.8621 nmol/L (4.2 µg/dL), indicating remission. Remission rates in adults are reported at 73% to 76% in selectively resected microadenomas and at 43% in macroadenomas [8], highlighting the need for regular follow-up visits to detect recurrence.

Following the surgery, the patient experienced diabetes insipidus, a relatively common postoperative occurrence, albeit usually transient [8]. It is recommended to monitor serum sodium levels during the first 5 to 14 days postsurgery for early detection and management. Additionally, pituitary deficiencies may manifest following surgery. In this patient, prolactin levels were compromised, potentially impacting sexual response. However, postoperative somatomedin levels were normal, and gonadotropins were not measured due to the patient's age group, as no additional clinical decisions were anticipated based on those results. Secondary hypothyroidism was diagnosed postoperatively.

Moving forward, it is important to emphasize certain clinical signs and symptoms for diagnosing CD. The combination of low bone mineral density (Likelihood Ratio [LR] +21.33), central obesity (LR +3.10), and arterial hypertension (LR + 2.29) [9] has a higher positive LR than some symptoms considered “characteristic,” such as reddish-purple striae, plethora, proximal muscle weakness, and unexplained bruising [2, 10]. It is essential to give relevance to the signs the patient may present, emphasizing signs that have been proven to have an increased odds ratio (OR) such as osteoporosis (OR 3.8), myopathies (OR 6.0), metabolic syndrome (OR 2.7) and adrenal adenoma (OR 2.4) [9‐11]. The simultaneous development and worsening of these conditions should raise suspicion for underlying issues. Understanding the evolving nature of CD signs highlights the importance of vigilance during medical examinations, prioritizing the diagnostic focus, and enabling prompt initiation of treatment.

Recognizing the overlap of certain clinical features in CS is fundamental to achieving a timely diagnosis.

## Learning Points

- CS diagnosis is challenging due to the absence of pathognomonic signs and symptoms and the overlap of features present in many pathologies, such as metabolic syndrome.
- Early detection of CS is crucial, given its association with high morbidity and mortality resulting from chronic exposure to glucocorticoids.
- Recognizing the combination of low bone mineral density, obesity, hypertension, and diabetes as valuable clinical indicators is key in identifying CS.
- Interdisciplinary collaboration is essential to achieve a comprehensive diagnostic approach.

## Data Availability

Restrictions apply to the availability of some or all data generated or analyzed during this study to preserve patient confidentiality or because they were used under license. The corresponding author will on request detail the restrictions and any conditions under which access to some data may be provided.

## Abbreviations

ACTH: adrenocorticotropic hormone
CD: Cushing disease
CRH: corticotropin-releasing hormone
CS: Cushing syndrome
HDDST: high-dose dexamethasone suppression test
HPA: hypothalamic-pituitary-adrenal
LDDST: low-dose dexamethasone suppression test
LNSC: late-night salivary cortisol
MRI: magnetic resonance imaging
OR: odds ratio
RV: reference value
UFC: urinary free cortisol

## References

[1] Hakami OA, Ahmed S, Karavitaki N. Epidemiology and mortality of Cushing's syndrome. Best Pract Res Clin Endocrinol Metab. 2021;35(1):101521. 33766428 10.1016/j.beem.2021.101521

[2] Nieman LK, Biller BMK, Findling JW, et al The diagnosis of Cushing's syndrome: an endocrine society clinical practice guideline. J Clin Endocrinol Metab. 2008;93(5):1526‐1540. 18334580 10.1210/jc.2008-0125 PMC2386281

[3] Gutiérrez Restrepo J, Latorre Sierra G, Campuzano Maya G. Síndrome de cushing. Med Lab. 2009;15:411‐430.

[4] Petersenn S, Newell-Price J, Findling JW, et al High variability in baseline urinary free cortisol values in patients with Cushing's disease. Clin Endocrinol (Oxf). 2014;80(2):261‐269. 23746264 10.1111/cen.12259 PMC4231220

[5] Lila AR, Sarathi V, Jagtap VS, Bandgar T, Menon P, Shah NS. Cushing's syndrome: stepwise approach to diagnosis. Indian J Endocrinol Metab. 2011;15(Suppl4):S317‐S321. 22145134 10.4103/2230-8210.86974 PMC3230095

[6] Dogra P, Vijayashankar NP. Dexamethasone suppression test. In: *StatPearls* StatPearls Publishing; 2024. Accessed January 29, 2024. http://www.ncbi.nlm.nih.gov/books/NBK542317/31194457

[7] Müller OA, Dörr HG, Hagen B, Stalla GK, von Werder K. Corticotropin releasing factor (CRF)-stimulation test in normal controls and patients with disturbances of the hypothalamo-pituitary-adrenal axis. Klin Wochenschr. 1982;60(24):1485‐1491. 6300509 10.1007/BF01716099

[8] Nieman LK, Biller BMK, Findling JW, et al Treatment of Cushing's syndrome: an endocrine society clinical practice guideline. J Clin Endocrinol Metab. 2015;100(8):2807‐2831. 26222757 10.1210/jc.2015-1818 PMC4525003

[9] Aron DC . Cushing's syndrome: why is diagnosis so difficult? Rev Endocr Metab Disord. 2010;11(2):105‐116. 20217244 10.1007/s11154-010-9127-3

[10] Braun LT, Vogel F, Zopp S, et al Whom should we screen for cushing syndrome? the Endocrine Society practice guideline recommendations 2008 revisited. J Clin Endocrinol Metab. 2022;107(9):e3723‐e3730. 35730067 10.1210/clinem/dgac379 PMC9387700

[11] Schneider HJ, Dimopoulou C, Stalla GK, Reincke M, Schopohl J. Discriminatory value of signs and symptoms in Cushing's syndrome revisited: what has changed in 30 years? Clin Endocrinol (Oxf). 2013;78(1):153‐154. 22775352 10.1111/j.1365-2265.2012.04488.x

